# Supplementary material for: Shared genetic and neuroimmune architecture links type 1 diabetes with neurocognitive traits
Source: Nat Commun. 2026 Mar 13;17:4057. doi: 10.1038/s41467-026-70694-8 (PMC13139607; doi:10.1038/s41467-026-70694-8)
Supplement: Supplementary file 1 — Supplementary Information [file 41467_2026_70694_MOESM1_ESM.pdf]

Shared genetic and neuroimmune architecture links type 1 diabetes with neurocognitive traits

Priscilla Saarah[1,2†], Zehra A. Syeda[1,2†], Ziang Xu[1,2], Yikai Dong[1,2], Habei Jiang[1,2], Michelle Shanguyhia[1,2], Sourav Roy[1,2], Biqing Zhu[3], Le Zhang[4,5], Andrew T. Dewan[6,7], Samira Asgari[8,9], David A. Alagpulinsa[1,2*]

1. Yale Center for Molecular & Systems Metabolism, Yale University School of Medicine, New Haven, CT, USA.
2. Department of Comparative Medicine, Yale University School of Medicine, New Haven, CT, USA.
3. Program of Computational Biology and Bioinformatics, Yale University, New Haven, CT 06510, USA.
4. Department of Neurology, Yale University School of Medicine, New Haven, CT, USA.
5. Department of Neuroscience, Yale University School of Medicine, New Haven, CT, USA.
6. Department of Chronic Disease Epidemiology, Yale School of Public Health, New Haven, CT, USA.
7. Center for Perinatal, Pediatric and Environmental Epidemiology, Yale School of Public Health, New Haven, CT, USA.
8. Institute for Genomic Health, Icahn School of Medicine at Mount Sinai, New York, NY, USA.
9. Department of Genetics and Genomic Sciences, Icahn School of Medicine at Mount Sinai, New York, NY, USA.

[†]These authors contributed equally.

*Corresponding author: David A. Alagpulinsa (david.alagpulinsa@yale.edu).

Supplementary Information

Supplementary Tables

No.	Trait / Dataset	Population	Sample size	PMID	Accession No/Reference
1	T1D	European	520 580 (18 942 cases)	34012112	GCST90014023
2	Bipolar disorder	European	413 466 (41 917 cases)	34002096	
3	ASD	European	46 350 (18 381 cases)	30804558	
4	Alzheimer’s disease	European	487 511 (85 934 cases)	35379992	GCST90027158
5	Parkinson’s disease	European	1 019 060 (37 688 cases)	31701892	GCST009324
6	Multiple sclerosis	European	38 589 (14 498 cases)	24076602	GCST005531
7	Myasthenia gravis	European	38 243 (1 873 cases)	35074870	GCST90093061
8	Major depression	European	92 957 (29 475 cases)	31969693	GCST009979
9	Intelligence	European	269 867	29942086	GCST006250
10	Education	European	405 072	27225129	GCST003676
11	Ischemic Stroke	European	1, 296, 908 (62, 100 cases)	36180795	GCST90104540
12	OCD	European	9 725 (2 688 cases)	28761083	
13	Migraine	European	513 266 (26 052 cases)	37415806	GCST90271641
14	Schizophrenia	European	130 644 (53 386 cases)	35396580	
15	Insomnia	European	2 365 010 (593 724 cases)	35835914	
16	Sleep duration	European	445 966	37770476	
17	ADHD	European	225 534 (38 691 cases)	36702997	
18	Neuroticism	European	393 411	29892013	GCST90029028
19	PTSD	European	1 222 882 (137 136 cases)	**38637617**	
20	ALS	European	138 086 (27 205 cases)	34873335	GCST90027164
21	Executive function	European	427 037	36150907	GCST90162547
22	SC-blood eQTL	European	982	35389779	[1]
23	Regional brain eQTL	European	838 (GTEx v8)	**29022597**	[2]
24	SC-brain eQTL	European	192	35915177	[3]
25	18 immune cell types	European	200	**27863251**	

Supplementary Table 1 | GWAS and eQTL datasets used in this study. List of genome-wide association studies (GWAS) and expression quantitative trait loci (eQTL) resources included in the analyses. All datasets were derived from individuals of predominantly European ancestry. GWAS covered T1D, cognitive and educational traits, psychiatric disorders, neurological diseases, and sleep phenotypes, with sample sizes ranging from tens of thousands to over two million participants. eQTL resources included bulk brain regions (GTEx v8), single-cell brain and blood, and purified immune cell types. For all datasets, we applied consistent quality control filters (autosomal SNPs, MAF >1%, INFO >0.9, allele harmonization, exclusion of ambiguous strands, and removal of the extended MHC region). Linkage disequilibrium was estimated using European reference panels from the 1000 Genomes Project Phase 3. T1D, type 1 diabetes; EXF, executive functioning; Sc, single cell; eQTL, expression quantitative trait loci; ASD, autism spectrum disorders; AD, Alzheimer’s disease; PD, Parkinson’s disease; MS, multiple sclerosis; MG, myasthenia gravis; OCD, obsessive-compulsive disorder; ADHD, attention deficit hyperactive disorder; PTSD, post-traumatic stress disorder; ALS, amyotrophic lateral

sclerosis; EDU, educational attainment; SC-blood eQTL, single-cell blood eQTL; SC-brain eQTL, single-cell brain eQTL.

REFERENCES

1. Yazar, S., et al., *Single-cell eQTL mapping identifies cell type-specific genetic control of autoimmune disease.* Science, 2022. **376**(6589): p. eabf3041.
2. Battle, A., et al., *Genetic effects on gene expression across human tissues.* Nature, 2017. **550**(7675): p. 204-213.
3. Bryois, J., et al., *Cell-type-specific cis-eQTLs in eight human brain cell types identify novel risk genes for psychiatric and neurological disorders.* Nat Neurosci, 2022. **25**(8): p. 1104-1112.

Trait	n_conjFDR_loci	Mean χ²	h²_obs	h²_obs_se	h²_Z
Bipolar disorder	13	1.5887	0.0716	0.0028	25.57
Multiple sclerosis	109	2.1596	1.5271	0.2520	6.06
Autism spectrum disorder	6	1.1998	0.2521	0.0217	11.62
Myasthenia gravis	69	1.0988	0.0095	0.0016	5.94
Educational attainment	45	1.6447	0.0918	0.0033	27.82
Executive function	21	1.8415	0.0931	0.0042	22.17
Insomnia	1	1.3659	0.0456	0.0021	21.71
Migraine	9	1.2492	0.0220	0.0015	14.67
Intelligence	63	2.0450	0.1883	0.0069	27.29
Alzheimer's disease	29	1.2735	0.0189	0.0031	6.10
Neuroticism	19	1.8629	0.1145	0.0041	27.93
Parkinson's disease	9	1.0893	0.0059	0.0006	9.83
ADHD	1	1.4490	0.0948	0.0045	21.07
ALS	6	1.1313	0.0382	0.0044	8.68

Supplementary Table 2 | GWAS power metrics and conjFDR pleiotropic locus counts for type 1 diabetes and neurocognitive trait pairs. This table summarizes genome-wide association study power metrics for each external trait paired with type 1 diabetes (effective sample size for T1D: n = 520,580; 18,942 cases), including mean χ^2 statistics and LD score regression SNP-heritability Z-scores. The number of loci jointly associated at conjFDR < 0.05 is shown for each trait pair. ALS, amyotrophic lateral sclerosis; ADHD, attention deficit/hyperactive disorder; T1D, type 1 diabetes

Supplementary Figures

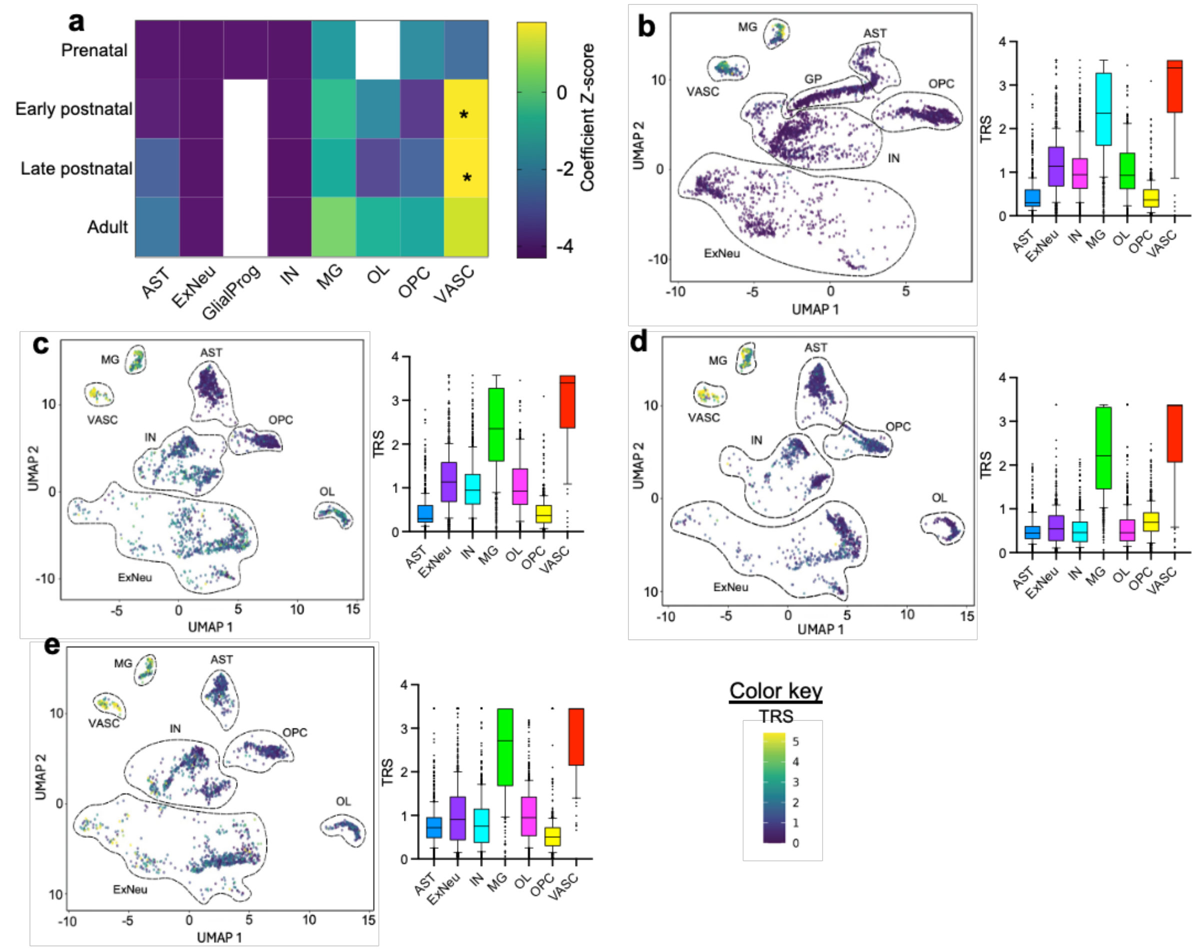

Supplementary Figure 1 | Enrichment of height heritability in brain-resident cell types across neurodevelopment. (**a**) Stratified LD score regression (S-LDSC) analysis of height genome-wide association study variants in accessible chromatin of brain cell types—astrocytes (AST), excitatory neurons (ExNeu), glial progenitors (GLIALPROG), inhibitory neurons (IN), microglia (MG), oligodendrocyte progenitor cells (OPC), and vascular cells (VASC)—across four neurodevelopmental stages (prenatal, early postnatal, late postnatal, and adult), profiled by single-nucleus assay for transposase-accessible chromatin sequencing. The heatmap displays S-LDSC τ Z-scores (τ/SE), reflecting cell-type–specific heritability enrichment. Asterisks indicate nominal significance based on one-sided P values ($P < 0.05$) from stratified LD score regression. Source data for panel (a) are provided in **Supplementary Data 10**. (**b–e**) SCAVENGE trait-relevance scores (TRS) for height-associated variants in single-nucleus assay for transposase-accessible chromatin sequencing data

across prenatal (**b**), early postnatal (**c**), late postnatal (**d**), and adult (**e**) stages. Box plots show the median (centre line), interquartile range (IQR; box), and whiskers extending to 1.5× IQR. For SCAVENGE analyses, *n* denotes individual nuclei from the source single-nucleus datasets. Source data for panels (**b–e**) are provided in **Supplementary Data 14**. Statistical analyses were performed using publicly available genome-wide association study summary statistics and single-cell reference datasets. The unit of analysis corresponds to individual study participants in the original genome-wide association studies and individual nuclei in the single-cell datasets as defined in the source studies. No biological or technical replicates were generated in this study.

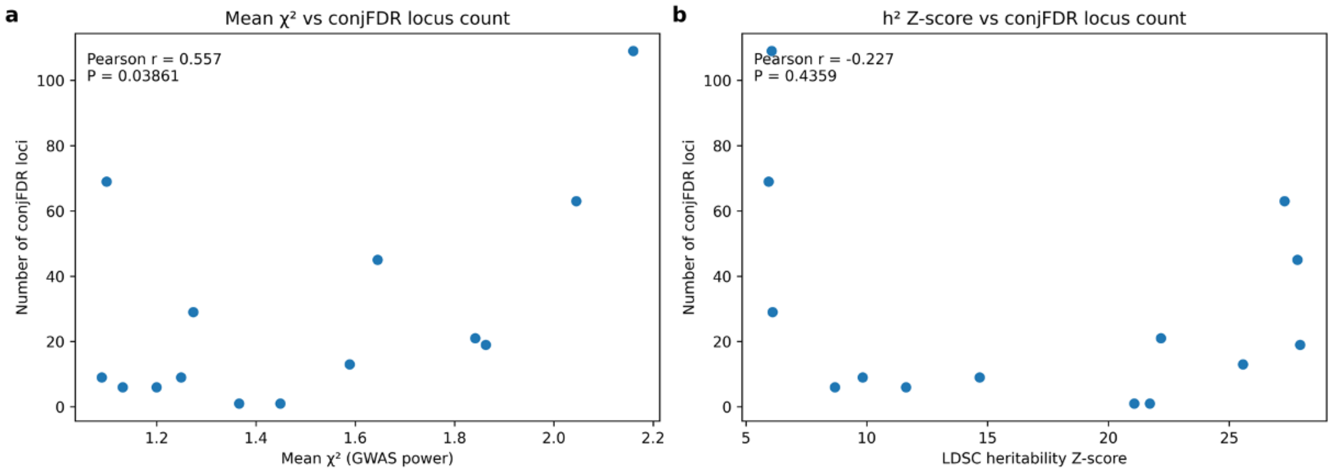

Supplementary Figure 2 | GWAS power versus pleiotropic locus discovery by conjFDR. Scatter plots showing the relationship between GWAS power and the number of loci jointly associated with type 1 diabetes (T1D) and each external neurocognitive or neuroimmune trait at conjFDR < 0.05. **(a)** Mean χ^2 statistic from LDSC for the external trait GWAS versus conjFDR pleiotropic locus count. **(b)** LDSC SNP-heritability Z-score for the external trait GWAS, computed as $Z(h^2)=h^2\_obs/SE(h^2\_obs)$, versus conjFDR pleiotropic locus count. Each point represents one external trait; Pearson correlation coefficients (r) and corresponding two-sided P values are shown.

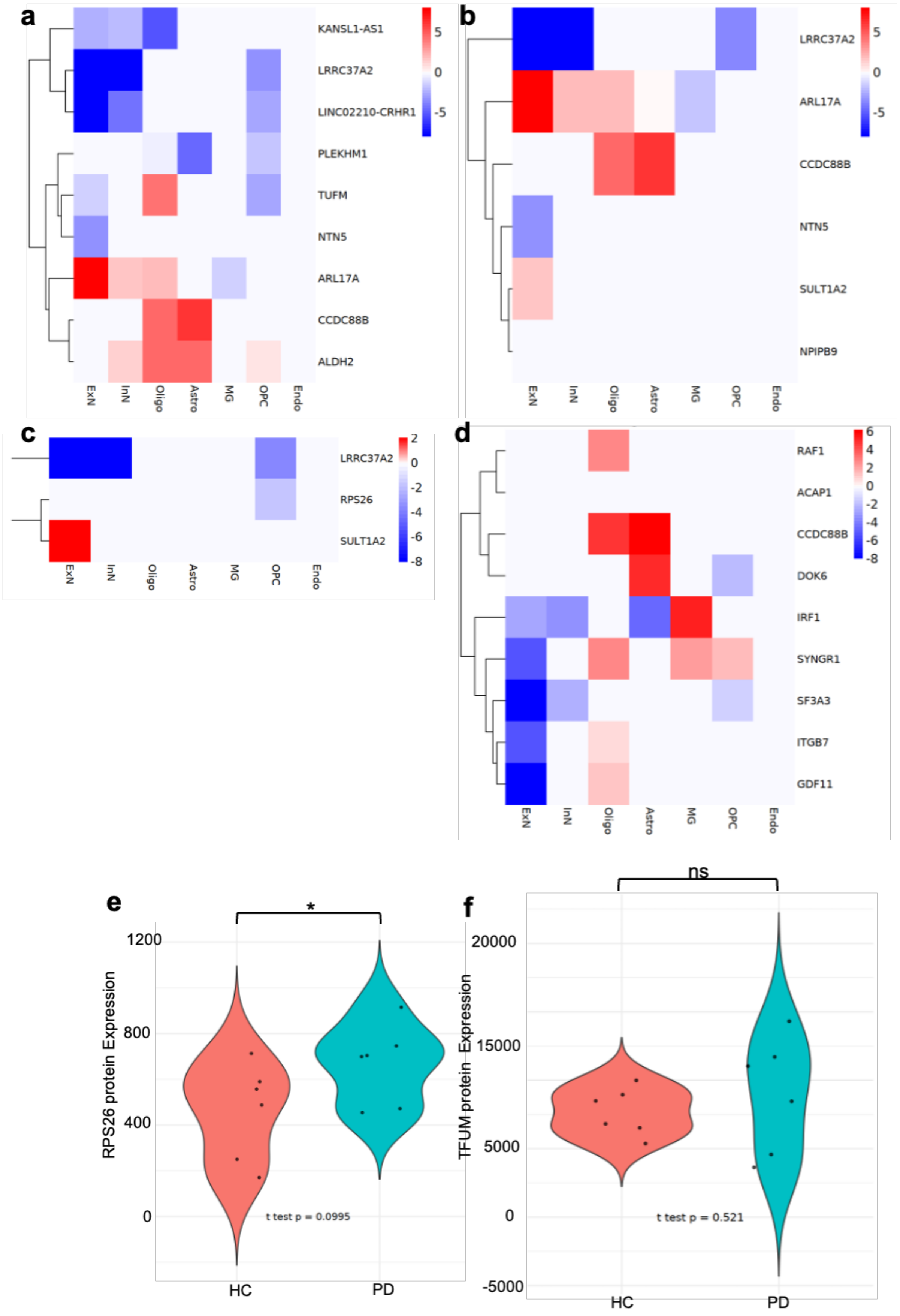

a
KANSL1-AS1
LRRC37A2
LINC02210-CRHR1
PLEKHM1
TUFM
NTN5
ARL17A
CCDC88B
ALDH2
5
0
-5
ExN
InN
Oligo
Astro
MG
OPC
Endo
b
LRRC37A2
ARL17A
CCDC88B
NTN5
SULT1A2
NPIPB9
5
0
-5
ExN
InN
Oligo
Astro
MG
OPC
Endo
c
LRRC37A2
RPS26
SULT1A2
2
0
-2
-4
-6
-8
ExN
InN
Oligo
Astro
MG
OPC
Endo
d
RAF1
ACAP1
CCDC88B
DOK6
IRF1
SYNGR1
SF3A3
ITGB7
GDF11
6
4
2
0
-2
-4
-6
-8
ExN
InN
Oligo
Astro
MG
OPC
Endo
e
*
RPS26 protein Expression
1200
800
400
0
t test p = 0.0995
HC
PD
f
ns
TFUM protein Expression
20000
15000
5000
0
-5000
t test p = 0.521
HC
PD

Supplementary Figure 3 | Differential transcriptomic and proteomic expression of SMR/HEIDI-identified genes in Parkinson's disease brain tissue. Genes whose genetically predicted expression in cortex (**a**), frontal cortex BA9 (**b**), substantia nigra (**c**), or microglia (**d**) were associated with type 1 diabetes risk in SMR/HEIDI analyses were evaluated for differential expression using publicly available single-nucleus RNA-seq datasets from Parkinson's disease (PD) and control prefrontal cortex (ref. 46). Each heatmap depicts normalized transcript expression (z-score) for PD versus control samples, with rows corresponding to genes and columns to individual samples. Statistical significance was assessed using DESeq2 with covariate adjustment for age, sex, and postmortem interval; only genes with adjusted $P < 0.05$ are shown. (**e–f**) Differential protein abundance of SMR-identified genes with brain-specific eQTL associations with type 1 diabetes, quantified using label-free mass spectrometry in the same PD and control cohort (ref. 46). Bars represent mean normalized protein abundance ± standard error. Significance was assessed using moderated t-tests with Benjamini–Hochberg correction. A relaxed threshold of $P < 0.1$ was used due to limited sample size; proteins marked with an asterisk (*) reached $P < 0.1$.
