# Supplementary material for: Shared genetic and neuroimmune architecture links type 1 diabetes with neurocognitive traits
Source: Nat Commun. 2026 Mar 13;17:4057. doi: 10.1038/s41467-026-70694-8 (PMC13139607; doi:10.1038/s41467-026-70694-8)
Supplement: Supplementary file 2 — Description of Additional Supplementary Files [file 41467_2026_70694_MOESM2_ESM.pdf]

Description of Additional Supplementary Files

Supplementary Data 1 | Genome-wide genetic correlations between type 1 diabetes (T1D) and neurocognitive traits estimated by LD score regression (LDSC). This table reports pairwise genome-wide genetic correlations (rg) between T1D and neurocognitive traits estimated using LDSC. For each trait pair, rg values, standard errors (SE), z-scores, and p values are shown. SNP-based heritability (h^2) estimates with corresponding SEs, as well as LDSC intercepts and cross-trait genetic covariance metrics, are also provided. All p values are two-sided unless otherwise specified.

Abbreviations: T1D, type 1 diabetes; AN, anorexia nervosa; BiP, bipolar disorder; ASD, autism spectrum disorder; AD, Alzheimer's disease; PD, Parkinson's disease; MS, multiple sclerosis; MG, myasthenia gravis; IS, ischemic stroke; ADHD, attention deficit/hyperactivity disorder; PTSD, post-traumatic stress disorder; ALS, amyotrophic lateral sclerosis; MDD, major depressive disorder; OCD, obsessive-compulsive disorder.

Supplementary Data 2 | Conjunctional false discovery rate (conjFDR) analyses of type 1 diabetes (T1D) and neurocognitive traits. This table contains multiple sheets, each reporting conjFDR results for T1D paired with a specific neurocognitive trait (for example, T1D_ASD, T1D_AD, and T1D_PD). Columns include SNP identifier, chromosome, genomic position, p values, and mapped genes. ConjFDR integrates genome-wide association study summary statistics across T1D and each trait to identify shared loci beyond single-trait significance thresholds. Reported loci represent putative pleiotropic signals highlighting regions of genetic overlap between T1D and neurocognitive traits. Loci surpassing the significance threshold (conjFDR < 0.05) are considered jointly associated with both traits.

Supplementary Data 3 | Bidirectional Mendelian randomization analyses between type 1 diabetes and neurocognitive traits. This table summarizes two-sample Mendelian randomization (MR) analyses evaluating bidirectional causal relationships between type 1 diabetes (T1D) and neurocognitive traits. In *T1D_exp_Neuro_out*, genetic liability to T1D was modeled as the exposure and individual neurocognitive traits as outcomes. In *Neuro_exp_T1D_out*, neurocognitive traits were modeled as

exposures and T1D as the outcome. MR estimates were obtained using inverse variance weighted (IVW) with multiplicative random effects, weighted median, and MR-Egger methods. For each analysis, the number of single-nucleotide polymorphism (SNP) instruments, odds ratios (ORs) with 95% confidence intervals (CIs), and corresponding two-sided P values are reported. The MR-Egger intercept and its P value are provided to assess horizontal pleiotropy. Statistically significant results are highlighted in bold within each sheet.

Supplementary Data 4 | Brain and immune expression quantitative trait loci showing significant SMR/HEIDI associations between type 1 diabetes and neurocognitive traits. This table lists brain and immune cell or tissue expression quantitative trait loci (eQTLs) exhibiting pleiotropic associations with both type 1 diabetes (T1D) and at least one neurocognitive trait. Associations were evaluated using summary data–based Mendelian randomization (SMR) with false discovery rate (FDR) correction (FDR < 0.05), followed by the HEIDI (heterogeneity in dependent instruments) test, in which non-significant heterogeneity (HEIDI $P > 0.05$) supports consistency with a shared causal variant rather than linkage. Reported fields include genomic locus, gene, tissue or cell type, eQTL effect estimates and P values, and corresponding SMR effect estimates and significance for T1D and neurocognitive traits.

Supplementary Data 5 | Brain-region expression quantitative trait loci showing significant SMR/HEIDI associations with type 1 diabetes. This table lists expression quantitative trait loci (eQTLs) from bulk brain regions that show significant associations with type 1 diabetes (T1D). Associations were evaluated using summary data–based Mendelian randomization (SMR) with false discovery rate (FDR) correction (FDR < 0.05), followed by the HEIDI (heterogeneity in dependent instruments) test, in which non-significant heterogeneity (HEIDI $P > 0.05$) supports consistency with a shared causal variant rather than linkage. Reported fields include genomic locus, gene, brain region, eQTL effect estimates and P values, and corresponding SMR and HEIDI statistics for T1D.

Supplementary Data 6 | Whole-blood expression quantitative trait loci showing significant SMR/HEIDI associations with type 1 diabetes. This table summarizes whole-blood expression quantitative trait loci (eQTLs) that exhibit significant associations with type 1 diabetes (T1D). Associations were evaluated using summary data–based Mendelian randomization (SMR) with false discovery rate correction (FDR < 0.05), followed by the HEIDI (heterogeneity in dependent instruments) test, in which non-significant heterogeneity (HEIDI P > 0.05) supports consistency with a shared causal variant rather than linkage. Reported fields include genomic locus, gene, tissue, eQTL effect estimates and P values, and corresponding SMR and HEIDI statistics for T1D.

Supplementary Data 7 | Immune cell–specific expression quantitative trait loci showing significant SMR/HEIDI associations with type 1 diabetes. This table lists immune cell–specific expression quantitative trait loci (eQTLs) that demonstrate significant associations with type 1 diabetes (T1D). Associations were tested using summary data–based Mendelian randomization (SMR) with false discovery rate correction (FDR < 0.05), followed by the HEIDI (heterogeneity in dependent instruments) test, in which non-significant heterogeneity (HEIDI P > 0.05) supports consistency with a shared causal variant rather than linkage. Reported fields include genomic locus, gene, immune cell type, eQTL effect estimates and P values, and corresponding SMR and HEIDI statistics for T1D.

Supplementary Data 8 | Single-cell brain expression quantitative trait loci showing significant SMR/HEIDI associations with type 1 diabetes. This table summarizes expression quantitative trait loci (eQTLs) from single-nucleus brain cell types that exhibit significant associations with type 1 diabetes (T1D). Associations were identified using summary data–based Mendelian randomization (SMR) with false discovery rate correction (FDR < 0.05) and filtered using the HEIDI (heterogeneity in dependent instruments) test, in which non-significant heterogeneity (HEIDI P > 0.05) supports consistency with a shared causal variant rather than linkage. Reported fields include genomic locus, gene, brain cell type, eQTL effect estimates and P values, and corresponding SMR and HEIDI statistics for T1D.

Supplementary Data 9 | Stratified LD score regression (S-LDSC) heritability enrichment of type 1 diabetes across single-nucleus brain cell types. This table reports partitioned heritability enrichment results for type 1 diabetes derived using stratified LD score regression across accessible chromatin annotations from single-nucleus brain cell populations spanning neurodevelopmental stages.

Supplementary Data 10 | Stratified LD score regression (S-LDSC) heritability enrichment of height across single-nucleus brain cell types. This table provides S-LDSC enrichment statistics for height across the same single-nucleus brain cell annotations used for type 1 diabetes, serving as a non-immune complex trait comparator.

Supplementary Data 11 | SCAVENGE trait relevance scores for type 1 diabetes across single-nucleus brain cells. This table contains SCAVENGE-derived trait relevance scores quantifying cell-level enrichment patterns for type 1 diabetes across single-nucleus brain cell populations spanning neurodevelopment stages.

Supplementary Data 12 | SCAVENGE trait relevance scores for Alzheimer's disease across single-nucleus brain cells. This table reports SCAVENGE-based single-cell enrichment metrics for Alzheimer's disease across the analyzed brain cell populations.

Supplementary Data 13 | SCAVENGE trait relevance scores for bipolar disorder across single-nucleus brain cells. This table summarizes SCAVENGE-derived cell-level enrichment patterns for bipolar disorder.

Supplementary Data 14 | SCAVENGE trait relevance scores for height across single-nucleus brain cells. This table provides SCAVENGE enrichment metrics for height across the same cellular landscape, serving as an additional comparator trait.
